# Photo-Oxidative Protection of Chlorophyll *a* in C-Phycocyanin Aqueous Medium

**DOI:** 10.3390/antiox9121235

**Published:** 2020-12-05

**Authors:** Ji-Eun Hong, Jae-Hyun Lim, Tae-Yoon Kim, Hwa-Yong Jang, Han-Bin Oh, Bong-Geun Chung, Seung-Yop Lee

**Affiliations:** 1 Department of Biomedical Engineering, Sogang University, Baekbeom-ro 35, Mapo-gu, Seoul 04107, Korea; jieunhong753@gmail.com (J.-E.H.); boy6635@naver.com (J.-H.L.); taeyoonkim83@gmail.com (T.-Y.K.); 2 Department of Chemistry, Sogang University, Baekbeom-ro 35, Mapo-gu, Seoul 04107, Korea; 0422jangg@naver.com (H.-Y.J.); hanbinoh@sogang.ac.kr (H.-B.O.); 3 Department of Mechanical Engineering, Sogang University, Baekbeom-ro 35, Mapo-gu, Seoul 04107, Korea

**Keywords:** chlorophyll *a*, antioxidant activity, photodegradation, phycocyanin, cytotoxicity

## Abstract

In this study, potential protection of chlorophyll *a* from illumination and oxidation-induced decomposition has been examined using C-phycocyanin (C-PC) aqueous medium. Photo-oxidation resistance of chlorophyll *a* was monitored in various aqueous media using ultraviolet-visible spectroscopy and direct-infusion atmospheric pressure chemical ionization mass spectrometry analysis. The spectroscopy results showed that chlorophyll *a* in C-PC medium experienced the lowest rate of conversion to its derivatives; thus, it was demonstrated that chlorophyll *a* was mostly intact in the C-PC medium. Furthermore, the C-PC treated with chlorophyll *a* showed the lowest concentrations of malondialdehyde, and chlorophyll *a* in C-PC medium did not cause serious damage to human liver cells in vitro after intensive illumination. Therefore, we propose a new method of protecting chlorophyll *a* from photodegradation and oxidation using C-PC aqueous medium.

## 1. Introduction

Chlorophyll *a*, the most abundant and most important photosynthetic pigment responsible for absorption of light and creation of energy, represents approximately 75% of the green pigments in green plants and perhaps an even larger amount in certain algae [1,2,3]. Chlorophyll *a* consists predominantly of a porphyrin ring with a central magnesium ion (Mg^2+^) and a C–20 mono-unsaturated esterified phytol tail. A symmetrical arrangement of four pyrole groups forms the basic ring structure, that is, porphine, to which a five-membered isocyclic ring is added to produce phorbin [4]. A subtle modification of this basic structure can cause chlorophyll *a* to be converted to a blue/green pigment with maximum absorbance at 660–665 nm [5,6].

*Chlorella vulgaris* is a species of single-cell green microalgae belonging to the phylum Chlorophyta, and it contains the green photosynthetic pigments chlorophyll *a* and *b* in its chloroplasts. Moreover, *C. vulgaris* contains the largest amount of chlorophyll among Chlorophyta [7]. Natural chlorophylls in microalgae are important sources of bioactive compounds for nutraceutical, pharmaceutical, and cosmeceutical applications. Chlorophyll extract is predominantly used as a potent scavenger of peroxyl radicals, and chlorophyll *a* and its derivatives also have considerable antioxidant properties. The chlorophyll content of green vegetables which are frequently consumed generally exceeds the concentrations of other bioactive pigments such as carotenoids by up to five-fold. This relatively high chlorophyll concentration contributes significantly to the complete pool of plant chemical compounds [6,8,9].

Previous studies reported that metallo-chlorophyll derivatives show significantly higher antioxidant activity than Mg^2+^-free chlorophyll derivatives [4,10,11]. Moreover, the magnesium ion is known to be easily replaced even by weak acids, which results in paler and dusky-colored pheophytin [4]. Heat treatment or acidification can also lead to discoloration of chlorophylls from green to brown in plant tissues. Such color loss is a result of the conversion of natural chlorophylls to its derivatives which lack Mg^2+^ such as pheophytins and pyropheophytins [11]. Furthermore, the presence and characteristics of the central metal ion may be responsible for antioxidant activity of chlorophylls and their derivatives, and it may be strongly correlated with the electron-donation ability of the conjugated porphyrin system [11]. Hoshina et al. [10] confirmed that chlorophylls are more potent antioxidants than their metal-free derivatives, and they further demonstrated the importance of the porphyrin ring for inhibiting lipid autoxidation.

The chemical structure of chlorophyll may degrade due to exposure to light and oxygen. Photodegradation of chlorophyll solutions due to ultraviolet and visible light can result in irreversible breakdown of chlorophylls, accompanied by the occurrence of a number of intermediate and final derivative products. Singlet oxygen is involved in chlorophyll degradation under light conditions, and photo-oxidation of the porphyrin ring structure ultimately leads to the formation of colorless products [12,13]. Previous studies examined potential methods to prevent or minimize chlorophyll photodegradation in order to improve green color. To stabilize chlorophyll, one option is to add an antioxidant; alternatively, the central magnesium ion can be replaced with divalent metal ions such as copper (Cu^2+^) and zinc (Zn^2+^) [4,14,15,16]. However, novel approaches to prevent chlorophyll degradation and to enhance the stabilization performance of chlorophyll molecules are required. In addition to the traditional use of chlorophyll derivatives in antioxidant medicine, these pigments are used for cancer prevention and potentially therapeutic agents since some chlorophyll derivatives can induce potential cellular photo-toxins and apoptosis in cancerous cells under intensive light conditions [11,17,18]. However, several studies have been conducted to elucidate the cancer-protection activity of natural antioxidants to protect cancer cells from apoptosis and enhance cell viability [19,20].

Chan et al. [18] reported the cell viability and anti-proliferative activity of normal human liver cells (WRL-68) and hepatocellular carcinoma cells (HepG2, Hep3B) by water-soluble pheophorbide *a*. Pheophorbide *a*, known as major antitumor component among chlorophyll derivatives, induced apoptosis in Hep3B cells but it was non-toxic in normal cells (WRL-68). In humans, the liver is the primary detoxifying organ that decomposes various compounds which would elicit oxidative stress [21]. Oxidative stress increases intracellular levels of reactive oxygen species (ROS) that can cause DNA damage, lipid peroxidation, hepatic stellate cell activation, and dysfunction of Kupffer cells in the liver [22]. Accumulation of ROS in hepatocytes is associated with various liver diseases such as steatosis, hepatitis, and liver fibrosis or cirrhosis [23,24]. To prevent such diseases, it is important to regulate ROS levels in the liver by administration of antioxidants such as uric acid, vitamin E, glutathione, and chlorophyll [21,25,26]. Chlorophyll *a* has several particular advantages as an antioxidant agent. It accounts for approximately 75% of the chlorophyll derivatives and can be extracted from various photosynthetic organisms [27,28]; however, chlorophyll *a* molecules are easily dissociated by exposure to light and oxygen.

Here, we propose the use of C-phycocyanin (C-PC) aqueous solution as a new medium to prevent the dissociation of chlorophyll *a* molecules. To the best of our knowledge, photo-stabilization of natural chlorophyll *a* using a C-PC aqueous medium has never been investigated before. In this study, water-soluble C-PC which is a photosynthetic assistant protein that can efficiently capture light energy was isolated from *Spirulina* sp. to examine its stabilization effects on the structure of chlorophyll *a*, which is known to exert strong protective effects against photodegradation under certain light conditions [29,30,31,32]. One of the objectives of this study was to evaluate the C-PC aqueous medium for protection of chlorophyll *a* from photodegradation using ultraviolet-visible (UV/VIS) spectroscopy and mass spectrometry. A malondialdehyde (MDA) assay was performed to evaluate whether chlorophyll *a* protected by C-PC under intense light conditions could exhibit antioxidative effects. Furthermore, we evaluated cytotoxic effects in human liver HepG2 cells treated with chlorophyll *a* and C-PC aqueous medium to investigate whether chlorophyll *a* and derivatives in C-PC enhance cell viability as antioxidants or promote antitumor effect in the liver cells. The investigated method may help preserve the intrinsic antioxidation property of chlorophyll *a* for longer periods of time and may, thus, facilitate its usage for nutraceutical, cosmetic, and pharmaceutical applications.

## 2. Materials and Methods

### 2.1. Reagents and Chemicals

*C. vulgaris* strain KMMCC-9 (UTEX-26) were provided by the Korea Marine Microalgae Culture Center, Busan, Korea. Pharmaceutical and food-grade C-PC powder (Rongsheng Biotechnology, Shaanxi, China), lyophilized powder of C-PC from *Spirulina* sp., chlorophyll *a* from spinach, 2-thiobarbituric acid, trichloroacetic acid, and linoleic acid were purchased from (Sigma-Aldrich, Seoul, Korea). All reagents were of analytical grade.

### 2.2. Extraction of Chloropyll a

*C. vulgaris* was cultivated in BG11 medium in a cell culture flask with a filter cap (SPL Life Sciences, Pocheon, Korea) inside an incubator (BioFree, Seoul, Korea) at 25 °C for 8–10 days. Cells were transferred to a 10-L container with a filling/venting closure ports cap (Nalgene, Rochester, NY, USA) and were grown at room temperature. Chlorophyll *a* and chlorophyll b are the predominant chlorophylls in plants, typically present in a ratio of 3:1. The chlorophyll *a* content and proportion can be increased during cultivation by adjusting light conditions [33,34,35,36]. In this experiment, cells were cultivated at a photoperiod of 16/8 h (light/dark) to enhance chlorophyll *a* content [33]. The increase in algal biomass was determined using a UV/VIS spectrophotometer (Genesys 10S, Thermo Fisher Scientific, Waltham, MA, USA) at 686 nm. One liter of algal cell culture (biomass) was transferred to a glass bottle which was then stored in a refrigerator (4 °C) for three days to let cells sediment by gravity. A condensed culture was collected and was placed in a 50-mL conical tube for centrifugation at 1008 RCF for 10 min. Cell pellets were then washed twice with 60% ethanol, and the supernatant was carefully aspirated. The cell pellets were re-suspended using 40 mL 99.9% ethanol followed by thorough vortex mixing for 20 s, and chlorophyll was collected after 24 h of incubation at 4 °C according to a previously described method [37]. Extracted chlorophyll contained mainly chlorophyll *a* rather than other chlorophyll derivatives as chlorophyll *a* is known to be the predominant fraction of pigment extracts from *C. vulgaris* [38] and the chlorophyll *a* proportion had been enhanced by the specific light conditions during cultivation [33].

### 2.3. Experimental Design

Sartory and Grobbelaar [39], performed a study on *Selenastrum capricornutum* and found that alcoholic solvents (ethanol (EtOH) and methanol) were preferable to other solvents (acetone and acetone with DMSO) for extraction of chlorophyll *a*. Thus, chlorophyll *a* extraction from *C. vulgaris* (30% [*v/v*]) was performed using EtOH, followed by dilution with either EtOH, deionized water (DW), or C-PC medium. The C-PC medium was prepared by dissolving C-PC powder (1 g) in 100 mL DW, and the solution was stirred continuously for 30 min at room temperature. The supernatant was filtered through a 0.22-µm syringe filter and stored at 4 °C before use. The solution was freshly prepared and further diluted, with some modifications [40]. After 4 h of intense light illumination, the remaining chlorophyll *a* was analyzed. To protect chlorophyll *a* from further light effects (photo-oxidation), the samples were covered using aluminum foil.

### 2.4. Identification of Chlorophyll a

Equal aliquots of chlorophyll *a* were added to each aqueous medium, and absorption spectra were measured using a UV/VIS spectrophotometer (Genesys 10S; Thermo Fisher Scientific, Waltham, MA, USA). All absorption spectra of chlorophyll *a* in each medium with continuous illumination treatment were scanned in a range of 250 to 800 nm.

Mass spectrometry experiments were performed on the chlorophyll extracts using the full mass scan mode of a triple quadrupole mass spectrometer (Quantiva, Thermo Fisher Scientific, Waltham, MA, USA) in positive-ion mode. An amount of chlorophyll *a* extracts (100 µL) in three different media, i.e., EtOH, DW, and C-PC medium, was further diluted using 900 µL methanol solvent with 1% formic acid, and then the mixtures were subjected to ionization by direct-infusion atmospheric pressure chemical ionization (APCI). The following mass spectrometric parameters were used in the experiments: Flow rate 20 μL/min; sheath gas (N_2_) pressure 25 psi; auxiliary gas (N_2_) pressure 5 psi; ion-transfer tube temperature 150 °C; vaporizer temperature 450 °C; and positive ion discharge current 4 μA. Xcalibur^TM^ v.4.1 software was used for data acquisition and processing.

### 2.5. Antioxidant Activity

A 10% (*v/v*) linoleic acid solution was diluted using 75% EtOH and 0.05 M phosphate buffer. Each sample containing the same amount of chlorophyll *a* in different media was added to 3 mL 10% linoleic acid, 3 mL DW, and 6 mL 0.05 M phosphate buffer mixture, and the volumes were adjusted to 15 mL using phosphate buffer. The samples were placed under 100 µmol∙m^−2^∙s^−1^ of intense light and subsamples were collected every 2 h by modifying a previously-described method [41].

Trichloroacetic acid (TCA; 20 g) was dissolved in 100 mL DW (20% *w/v*), whereas 670 mg 2-thiobarbituric acid (TBA) was dissolved in 80 mL DW, which was then heated to 50 °C for 45 min in a water bath, and the volume was adjusted to 100 mL. The TBA solution was freshly prepared each time [42]. Three milliliters of each sample was mixed with 0.5 mL 20% TCA and 0.25 mL 0.67% TBA, and the mixtures were then heated to 98 °C for 10 min. After complete cooling to room temperature, the samples were centrifuged at 1008 RCF for 10 min. The supernatant was measured using a UV/VIS spectrophotometer at 532 nm. MDA has been identified as a product of lipid peroxidation that reacts with TBA to produce a red species with maximum absorption at 532 nm against a blank containing all the reagents except the lipid [43,44,45,46].

### 2.6. Cell Viability Assay

A cell counting kit-8 (CCK-8; Dojindo, Kumamoto, Japan) was used to analyze cell viability. Hepatocellular carcinoma HepG2 cells were seeded at 1 × 10^4^ cells in a 96-well plate with containing Gibco^TM^ RPMI 1640 medium (Thermo Fisher Scientific, Waltham, MA, USA) supplemented with 10% fetal bovine serum. After incubation for 24 h, HepG2 cells were treated with chlorophyll for 12 h in a 96-well plate. Each well plate was washed with Dulbecco’s phosphate-buffered saline, and 100 μL RPMI 1640 medium was subsequently added to the HepG2 cell cultures on the plates. Ten microliters CCK-8 solution was added to the 96-well plates which were then incubated for 1 h. Cell viability was analyzed using a microplate reader (iMark^TM^ Microplate Reader; Bio-RAD, Hercules, CA, USA) at 450 nm.

### 2.7. Statistical Analysis

All experiments were conducted in triplicates. An analysis of variance (ANOVA) was employed for statistical analyses using SPSS software v25 (IBM, Armonk, NY, USA); statistical significance is reported at *p* < 0.05. One-way ANOVA followed by Tukey’s multiple comparison test was used when more than one categorical independent variable was tested (such as MDA measurements over time). Differences in cell viability were tested using Student’ s *t*-test.

## 3. Results and Discussion

### 3.1. Stability of Chlorophyll a

**Figure 1:**
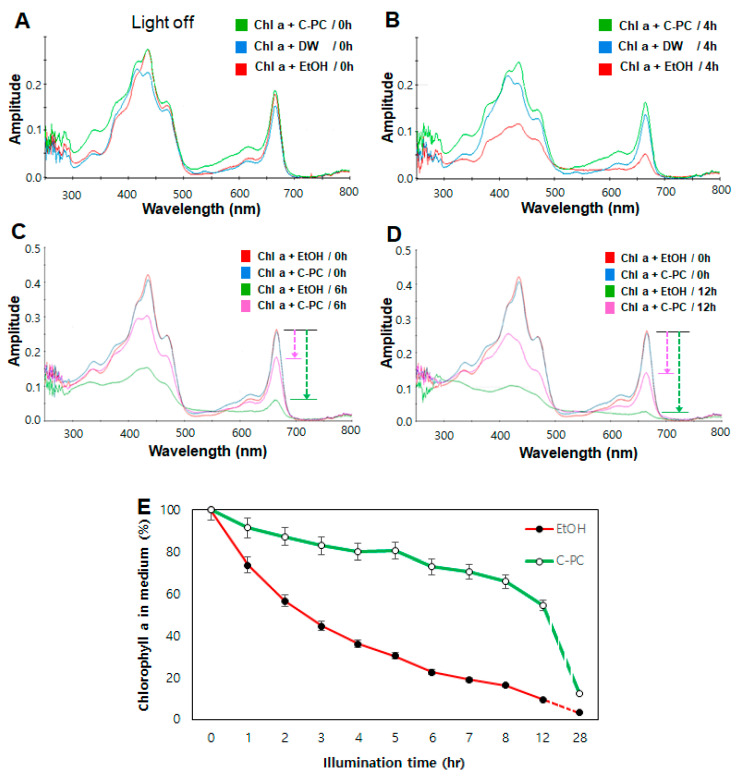
UV/VIS absorption spectra of chlorophyll *a* in ethanol (EtOH), deionized water (DW), and C-phycocyanin (C-PC) media. (**A**) Before light treatment. (**B**) After 4 h of intense white light illumination (100 µmol∙m^−2^∙s^−1^). The initial chlorophyll extracts added to EtOH, DW, or C-PC medium were prepared as described earlier. (**C**) UV/VIS absorption spectra of chlorophyll *a* in high C-PC solution (600 ppm) was compared after 6 h of illumination. (**D**) After 12 h of illumination (100 µmol∙m^−2^∙s^−1^). (**E**) Chlorophyll content in EtOH and C-PC solution per hour during illumination for 28 h. The values are expressed as means ± standard deviation (*n* = 3).

Quantification of chlorophyll *a* using UV/VIS spectroscopy depends on the choice of sample, solvent system, and spectrophotometer used [47]. UV/VIS absorption spectra of chlorophyll *a* extracted from *C. vulgaris* in various media, namely, EtOH, DW, and C-PC solution were acquired after continuous illumination with intense white light-emitting diodes (Stech LED, Seoul, Korea) at 100 µmol∙m^−2^∙s^−1^ (Figure 1). UV/VIS absorption spectra with two typical peaks in the blue and red ranges were recorded hourly during continuous illumination to examine chlorophyll *a* in the three cases with and without illumination. The absorption maxima of extracted chlorophyll strongly depend on the type of solvent, and with increasing solvent polarity, the red absorption maximum shifts from 660 to 665 nm and the blue absorption peak from 428 to 432 nm [47].

As shown in Figure 1A,B the UV/Vis light absorption of chlorophyll *a* (400 ppm) decreased significantly in the EtOH medium when the solution was illuminated for 4 h with intense white light, whereas absorption in other media, particularly in the C-PC solution (300 ppm), decreased to a lesser extent. After 4 h of illumination, the chlorophyll *a* content in EtOH medium as judged by the corresponding UV/VIS peak area at 664 nm decreased to 66.3%. In the C-PC solution, a high fraction of chlorophyll *a* remained stable.

For comparison, UV/VIS absorption spectra were obtained at higher concentrations of chlorophyll *a* (800 ppm) and C-PC solution (600 ppm) hourly after illumination with intense white light (see Figure 1C–E). When the illumination period was increased to 12 h, residual chlorophyll *a* was almost undetectable in the EtOH medium (Figure 1D), whereas the C-PC solution contained higher amounts of residual chlorophyll *a*. Figure 1E shows the residual chlorophyll *a* content in the EtOH and C-PC solutions, which were measured every hour during the 28-h illumination period using an established equation to evaluate pigment concentration [37,45]. In the EtOH medium, the concentration of chlorophyll *a* decreased rapidly from the beginning of the light treatment. The maximum residual content (54.5%) remained in the C-PC medium even after 12 h of illumination, whereas only a small fraction of chlorophyll *a* (9.7%) was detected in EtOH under the same illumination conditions. With the illumination period increasing from 12 to 28 h, the residual chlorophyll *a* gradually decreased to 12.4%. The residual chlorophyll *a* content in DW was slightly lower than that in the C-PC solution (data not shown). These results suggested that the stability of chlorophyll increased in the C-PC medium compared with that in EtOH medium; therefore, this stabilized chlorophyll *a* in the C-PC solution may be effectively used in any aqueous medium.

**Figure 2:**
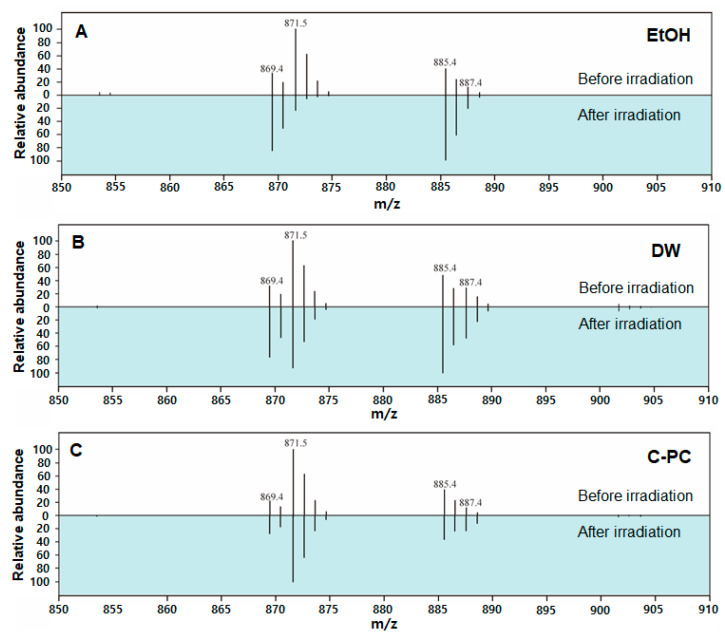
APCI-MS spectra acquired in the *m/z* range of 850 and 910 before (top) and after (bottom) intense light illumination of extracts from *Chlorella vulgaris* in three different media, namely, (**A**) EtOH, (**B**) DW, and (**C**) C-PC. Mass spectra were acquired using the centroid mode.

**Figure 3:**
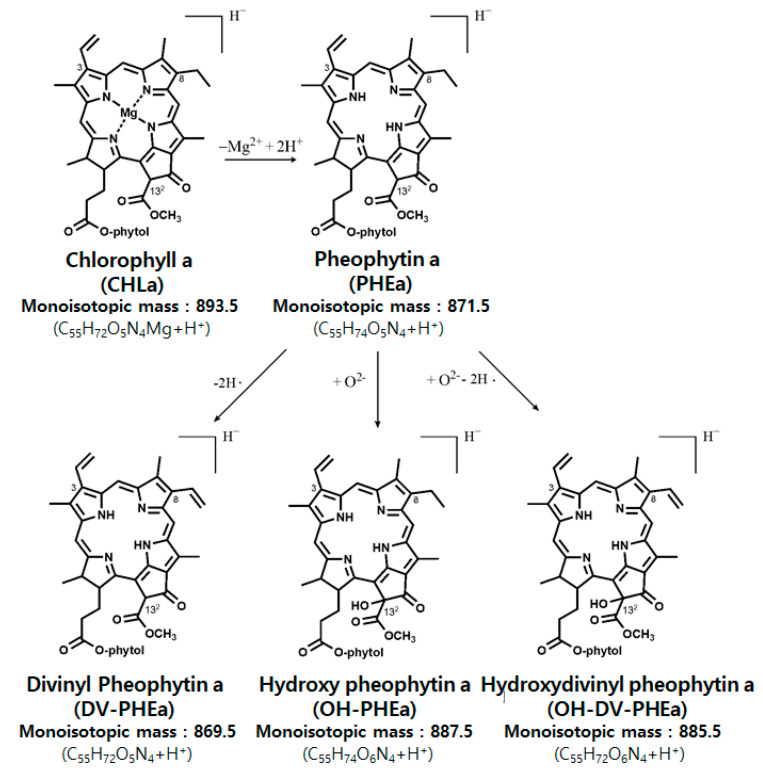
Structures and monoisotopic masses of the chlorophyll *a* derivatives.

Further analysis of the residual chlorophyll *a* content in each medium was assessed using direct-infusion APCI-MS experiments [48]. Figure 2 shows APCI-MS spectra acquired in the *m/z* range of 850–910 before (top) and after (bottom) 4 h light illumination of extracts from *C. vulgaris* in three different media, namely, EtOH, DW, and C-PC. In this *m/z* range, four peaks occurred at 869.5, 871.5, 885.5, and 887.5 in ascending order (note that the peaks indicate monoisotopic peaks of the corresponding molecular species ), and these peaks were assigned to divinyl pheophytin *a* (DV-PHEa), pheophytin *a* (PHEa), hydroxydivinyl pheophytin *a* (OH-DV-PHEa), and hydroxy pheophytin *a* (OH-PHEa), respectively, based on the results of previous studies [13,49,50] (see Table 1 and Figure 3). In APCI-MS spectra, molecular species with Mg^2+^ such as chlorophyll *a*, divinyl chlorophyll *a*, and hydroxydivinyl chlorophyll *a* were not detected as Mg^2+^ is rapidly removed under the applied acidic APCI-MS conditions. Instead, chlorophyll *a* and divinyl chlorophyll *a* were detected in the forms of PHEa and DV-PHEa, respectively. Furthermore, hydroxy chlorophyll *a* and hydroxydivinyl chlorophyll *a* were detected as OH-PHEa and OH-DV-PHEa, respectively.

Before intense light illumination, a multitude of chlorophyll *a* derivatives such as DV-PHEa (*m/z* 869.5), PHEa (*m/z* 871.5), OH-DV-PHEa (*m/z* 885.5), and OH-DV-PHEa (*m/z* 887.4) were observed, which suggested the presence of divinyl chlorophyll *a* (*m/z* 891.5), chlorophyll *a* (*m/z* 893.5), hydroxy chlorophyll *a* (*m/z* 909.5), and hydroxydivinyl chlorophyll *a* (*m/z* 907.5), respectively. Among these, the peak at *m/z* 871.5 was highest, followed by the peaks at *m/z* 885.5, 869.5, and 887.5. As shown in Figure 2 in the EtOH medium, after light illumination, a substantial amount of PHEa (*m/z* 871.3) was converted to DV-PHEa (*m/z* 869.5), OH-DV-PHEa (*m/z* 885.5), and OH-PHEa (*m/z* 887.5); thus, dehydrogenation and hydroxylation occurred substantially. In the DW medium, dehydrogenation and hydroxylation seemed to also have occurred, but to a lesser extent as compared with that in EtOH medium (Figure 2). In contrast, in the C-PC medium, dehydrogenation and hydroxylation occurred to negligible extent (Figure 2). More specifically, PHEa was found to decrease in EtOH, DW, and C-PC media by 95%, 45%, and 7%, respectively. These observations are in corroboration with the observed UV/VIS absorption spectra (Figure 1).

### 3.2. Determination of the Antioxidant Activity

There are two major mechanisms associated with the antioxidant effects of chlorophylls: Free-radical-scavenging activity and metabolic detoxification pathways [51]. Reactive oxygen species (ROS) occur within cells in various forms such as superoxide (O_2_•^−^ ), hydroxyl (•OH), peroxyl (ROO•), alkoxyl (RO•), hydroperoxyl (HO_2_•), hydrogen peroxide (H_2_O_2_), and singlet oxygen (^1^O_2_). Numerous studies have reported the antioxidant functions of chlorophylls [52,53]. The common experimental approach to assess ROS in different tissues of experimental animals (such as mice and rats) is to administer diets rich in copper chlorophyllins [53]. Furthermore, the results of some studies demonstrated the capacity of chlorophylls to reduce overall ROS levels in vitro [53,54,55]. The antioxidant ability of chlorophyll *a* derivatives as radical quenchers was revealed to be more effective than that of chlorophyll b; however, metal-free catabolites such as pheophytins and pyropheophytins showed 25%–30% lower antioxidant activity than metal derivatives such as chlorophylls and chlorophyllins [56].

To determine antioxidative effects according to the degree of chlorophyll *a* and pheophytin *a* stabilization, we performed MDA assays. Linoleic acid compounds or lipid components in biological living membranes begin to oxidize when hydrogen atoms are deprived of radicals or oxygen in the surroundings. Substances obtained from oxidation of lipids include alkoxy radicals, peroxyl radicals, hydroxyl radicals, MDA, and 4-hydroxymomenal. Among these, MDA is a representative low-molecular weight activating compound, and even in trace amounts, it binds to TBA to form a red compound. Experimental quantification of MDA is simple, and its abundance is considered a physiological marker of oxidative stress [6].

**Figure 4:**
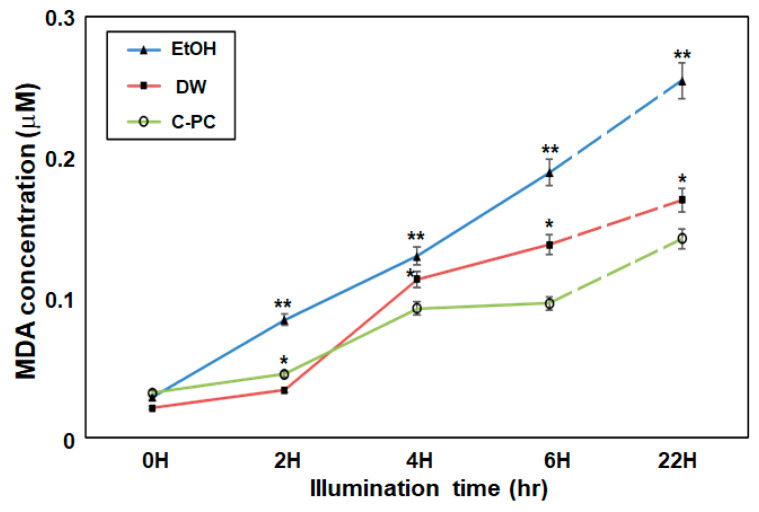
Changes in malondialdehyde (MDA) concentrations of chlorophylls in different media (EtOH, DW, and C-PC) during intense light illumination for 22 h. Linoleic acid was used as a substrate, and the antioxidant effects depending on the amount of chlorophyll *a* stabilized under intense light illumination conditions. All experiments were performed in triplicates the mean values of which are shown. Error bars indicate standard deviations; asterisks indicate significant differences between media (one-way-ANOVA with Tukey’s multiple comparison test, * *p* < 0.05, ** *p* < 0.01).

Linoleic acid was used as a lipid-rich model, and lipid oxidation was induced by intense light illumination of each reaction mixture. One molecule of MDA reacts with two molecules of TBA to form a red-colored, strongly visible light-absorbing (532 nm) and fluorescent derivate [43,44,45]. MDA concentrations in each medium under continuous lighting at an intensity of 100 µmol∙m^−2^∙s^−1^ were measured using the TBA assay described in Section 2.4. Initially, MDA levels were in the 0.02–0.03 µmol range in the three media (Figure 4). After 2 h, in the EtOH group, MDA accumulation began to substantially increase as chlorophyll breakdown occurred. After 4 h, photo-oxidation of chlorophyll was apparent in DW. In contrast, chlorophyll *a* in the C-PC solution showed strong anti-lipid peroxidation activity against photo-oxidation. Compared to DW and EtOH, C-PC showed 33% and 41% lower levels and 41% and 53% lower accumulation after 4 h and after 6 h, respectively (Figure 4). In corroboration with the results of previous studies [52], it was also demonstrated that chlorophyll stabilized by C-PC consistently exerted antioxidant effects and could maintain the color of chlorophyll *a* in aqueous medium. Continuous exposure to light for 22 h, chlorophyll *a* in C-PC medium also began to sustain damage and MDA accumulation increased. Thus, the degree of photo-stabilization of chlorophyll by the C-PC medium under continuous stress conditions is limited; however, photo-oxidative protection of chlorophyll has been confirmed after light exposure for more than 6 h regarding MDA accumulation. The batch spectrophotometric MDA method is a commonly used assay to measure lipid peroxidation; however, it lacks specificity because various nonlipid compounds can react with TBA and interfere with MDA analysis [46,57]. Nonetheless, this TBA-based MDA measurement is useful in the comparative analysis of antioxidant activity in various aqueous media.

In general, the central magnesium ion of chlorophyll is replaced by copper or zinc ions to stabilize chlorophyll; however, the copper-complex chlorophyll derivative (chlorophyllin) is dangerous if ingested at high doses [56]. The proposed C-PC medium, rather than the metal substitution method, may be used for maintaining the antioxidative function of chlorophyll *a* for longer periods of time.

### 3.3. Cell Viability Assay

As aforementioned, antioxidants have widely been used for protection of cells from damage by ROS [11,52]. However, some previous studies have reported contradictory effects of natural antioxidants on cancerous cells. One is the cancer preventative activity and potentially therapeutic agents of chlorophyll derivatives [11,18,55], and the other effect of natural antioxidants is the protection of cancer cells from apoptosis [19,20]. Therefore, we evaluated cytotoxic effects in human liver HepG2 cells treated with chlorophyll *a* in C-PC aqueous medium to investigate whether chlorophyll *a* and derivatives in C-PC enhances cell viability as antioxidants or causes antitumor effect on the cancerous cells.

**Figure 5:**
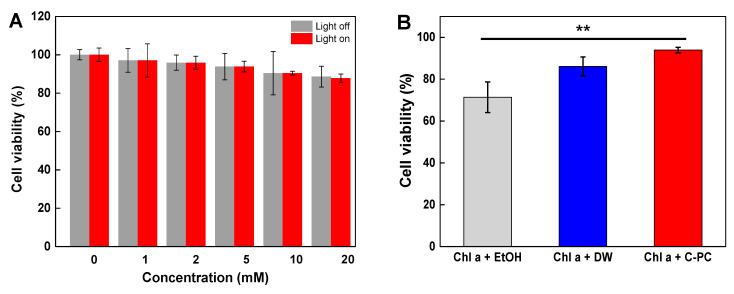
Viability of HepG2 cells treated with (**A**) various concentrations of only C-PC and (**B**) Chlorophyll *a* (Chl a, 20 mM) in EtOH, DW, and C-PC for 12 h with illumination (** *p* < 0.01).

Our experimental results showed that C-PC molecules could not only suppress the photodegradation via light illumination, but also degenerate the dehydrogenation and hydroxylation in an aqueous solution. To prove this phenomenon through in vitro study, chlorophyll *a* dissolved in EtOH, DW, and C-PC medium were treated with HepG2 cells and cell viability was analyzed using a CCK-8 assay (Figure 5). First, we tested the cytotoxicity in the cells treated with various concentrations of C-PC with or without illumination for 12 h (Figure 5A). Cell viability remained above 85%, indicating that C-PC was non-toxic at the used concentrations.

We subsequently evaluated the viability of cells treated with chlorophyll *a* in EtOH, DW, and C-PC (20 mM) with illumination (Figure 5B). We observed that the viability of the cells treated with chlorophyll *a* in EtOH and DW was 71% and 86%, respectively. This result corresponds to the high levels of dehydrogenation and hydroxylation produced by photo-degradation, resulting in cancer therapeutic effect [58]. On the other hand, the viability of the cells treated with chlorophyll *a* in C-PC medium was slightly increased (93%) as compared to control groups (chlorophyll *a* in EtOH or DW). Unlike the positive case of chlorophyll derivatives as antitumor agents, chlorophyll *a* in C-PC with illumination enhanced cell viability of the liver cancer cells as antioxidants, as shown in Figure 5B.

It is well known that pheophorbide *a* is the major antitumor component among chlorophyll derivatives [11,18]. Pheophorbide *a* is one of the significant degradation products of chlorophyll *a* and it can be formed by the removal of a magnesium ion and a phytyl group in chlorophyll *a*. Chan et al. [18] reported the cytotoxicity tests to investigate the anti-proliferative effect of pheophorbide *a* using normal liver cells (WRL-68) and hepatocellular carcinoma cells (HepG2, Hep3B). For WRL-68 cells treated with 20 µg/mL pheophorbide *a*, the cell survival rate is over 90% after 48 h of incubation. However, liver cancer cells showed lower cell viability (21% in HepG2 and 26% in Hep3B cells). As confirmed by APCI-MS spectra data in Figure 2, protection of chlorophyll *a* in C-PC from photo-degradation causes the lower decomposition into antitumor-related chlorophyll derivatives. Therefore, the enhanced cell viability of HepG2 cells in C-PC in Figure 5B, could be resulted from the antioxidant effect of chlorophyll *a* and its derivatives rather than the antitumor agents.

## 4. Conclusions

Chlorophyll *a* extracted from *C. vulgaris* in three different media was examined to confirm delayed degradation of chlorophyll *a* in C-PC medium under intense illumination using UV/VIS spectra and APCI-MS analysis. The MDA levels from the TBA assay showed that antioxidant activities of chlorophyll *a* in C-PC medium were superior to those of chlorophyll *a* in EtOH and DW because of the highest levels of residual chlorophyll *a* in C-PC solution after 4–6 h. These results suggest that chlorophyll *a* was stabilized in the form of pheophytins, which could produce evident antioxidative effects. An in vitro study on HepG2 cells demonstrated that viability of cells treated with chlorophyll *a* in C-PC was increased compared to those of controls (chlorophyll *a* in EtOH and DW). This suggested that C-PC molecules in liver cells inhibited illumination-induced oxidation and degradation of chlorophyll *a*. Consequently, the observed antioxidant activities may add value to the use of stabilized chlorophyll *a* in an aqueous medium as a bioactive compound for nutraceutical, cosmetic, and pharmaceutical applications.

## Figures and Tables

**Table 1:** Definition of the abbreviations of chlorophyll *a* derivatives shown in Figure 2.

No.	Name	Molecular Formula	Abbreviation	[M + H]^+^
1	Divinyl pheophytin *a*	C_55_H_72_O_5_N_4_ + H^+^	DV-PHEa	869.5
2	Pheophytin *a*	C_55_H_74_O_5_N_4_ + H^+^	PHEa	871.5
3	Hydroxydivinyl pheophytin *a*	C_55_H_72_O_6_N_4_ + H^+^	OH-DV-PHEa	885.5
4	Hydroxy pheophytin *a*	C_55_H_74_O_6_N_4_ + H^+^	OH-PHEa	887.5
